# Towards the integration, annotation and association of historical microarray experiments with RNA-seq

**DOI:** 10.1186/1471-2105-14-S14-S4

**Published:** 2013-10-09

**Authors:** Shweta S Chavan, Michael A Bauer, Erich A Peterson, Christoph J Heuck, Donald J Johann

**Affiliations:** 1 Myeloma Institute for Research and Therapy, University of Arkansas for Medical Sciences, Little Rock, AR, USA

## Abstract

**Background:**

Transcriptome analysis by microarrays has produced important advances in biomedicine. For instance in multiple myeloma (MM), microarray approaches led to the development of an effective disease subtyping via cluster assignment, and a 70 gene risk score. Both enabled an improved molecular understanding of MM, and have provided prognostic information for the purposes of clinical management. Many researchers are now transitioning to Next Generation Sequencing (NGS) approaches and RNA-seq in particular, due to its discovery-based nature, improved sensitivity, and dynamic range. Additionally, RNA-seq allows for the analysis of gene isoforms, splice variants, and novel gene fusions. Given the voluminous amounts of historical microarray data, there is now a need to associate and integrate microarray and RNA-seq data via advanced bioinformatic approaches.

**Methods:**

Custom software was developed following a model-view-controller (MVC) approach to integrate Affymetrix probe set-IDs, and gene annotation information from a variety of sources. The tool/approach employs an assortment of strategies to integrate, cross reference, and associate microarray and RNA-seq datasets.

**Results:**

Output from a variety of transcriptome reconstruction and quantitation tools (e.g., Cufflinks) can be directly integrated, and/or associated with Affymetrix probe set data, as well as necessary gene identifiers and/or symbols from a diversity of sources. Strategies are employed to maximize the annotation and cross referencing process. Custom gene sets (e.g., MM 70 risk score (GEP-70)) can be specified, and the tool can be directly assimilated into an RNA-seq pipeline.

**Conclusion:**

A novel bioinformatic approach to aid in the facilitation of both annotation and association of historic microarray data, in conjunction with richer RNA-seq data, is now assisting with the study of MM cancer biology.

## Background

Cancer diagnostics are now being revolutionized by advances and insights from biotechnology, computational science, and molecular biology. Molecular profiling approaches to both discover and better define individual patterns of disease related molecules is a principle requirement of *precision medicine *[1]. By revealing the molecular taxonomy of a patient's tumor, a precise and rational approach to treatment may be applied. This allows for customized therapeutic regimens versus categorical assignments. Improved therapeutic efficacies along with the minimization of toxicity are the principle aims [2,3].

Multiple Myeloma (MM) is a cancer of the bone marrow and is characterized by a malignant proliferation of plasma cells. Clinically, MM is typified by osteolytic bone lesions, anemia, hypercalcemia, and renal failure [4,5]. There are approximately 20,000 new cases and 10,000 deaths estimated to occur in the United States each year [6]. MM is generally considered incurable, with a clinical course characterized by remissions and relapses [7]. Patient survival has improved dramatically in the past 10-15 years, due to scientific and technological advances, which have enabled an improved understanding of the cancer biology. This has led to two new classes of medications, (Immunomodulatory drugs (IMiDs), e.g., thalidomide, lenalidomide, pomalidomide; and proteasome inhibitors, e.g., bortezomib, carfilzomib), new therapeutic approaches (e.g., autologous tandem transplant), and advances in cancer-based supportive care (e.g., bisphosphonates for bone metastasis) [8-10].

Microarray-based transcriptome approaches have resulted in: i) disease subtyping for MM, ii) the establishment of a 70 gene prognostic risk score (GEP-70) and, iii) the identification of therapeutic drug targets (e.g., *DKK1*) [11-14]. An improved understanding of MM on a molecular level has been achieved by these three accomplishments. Additionally, they have provided much needed prognostic information. Such information serves to assist with clinical management in settings of initial diagnosis, relapse, and also therapeutic assignments.

A commercial transcriptome-based molecular profiling assay for multiple myeloma does now exist. It is marketed by Signal Genetics (http://www.myelomahealth.com). The assay is called "MyPRS Plus" and is based on microarray technology. Certainly, if this assay is used in a phase III clinical trial, prognostication abilities may be appropriately evaluated.

By far the most mature use of advanced clinical assays, and molecular profiling in particular, is in breast cancer. Transcriptome-based molecular methods have resulted in two key assays, MammaPrint and Oncotype DX^®^, which are now contained in the National Comprehensive Cancer Network^® ^(NCCN^®^) guidelines [15,16]. MammaPrint is a 70 gene assay and has been approved by the FDA to assist with the assessment of recurrence of disease for women younger than 61 with certain types of breast cancer. Oncotype DX is a 21 gene assay for hormone receptor positive, axillary lymph node negative breast cancer, and quantifies the risk of recurrence as a continuous variable. The clinical question being addressed by OncoType DX is, if chemotherapy is given, will it be beneficial. Both assays are used in the prognostication of certain breast cancers [17,18]. The use of gene-expression signatures empowers the identification of specific subclasses of breast cancer. For over seven years, both assays have been used, and have displayed significant clinical utility [19]. Finally, clinical trials have been utilized to critically assess the effectiveness of these assays. [20-23]

Many researchers are now transitioning to Next Generation Sequencing (NGS) approaches and RNA-seq in particular, due to its discovery-based nature, improved sensitivity, and dynamic range. Table 1 is adapted from Wang, et. al.,[24] and showcases the major differences between microarray and RNA-seq technologies. Compared to microarray technologies, generally RNA-seq requires less mRNA, has single base resolution, and is able to distinguish allelic expression, splice isoforms, and discover new genes. Strandedness can also be accommodated, which improves specificity during data analysis. Compared to microarray analysis the computational requirements for RNA-seq are much more complex. The term "cost" refers to financial cost, and reflects computational hardware including infrastructure, as well as the time and expertise for data analysis. The correlation between microarray and RNA-seq data is problematic primarily due to dynamic range differences [25].

**Table 1:** Microarray vs. RNA-seq

	Microarray	RNA-seq
Principle	Hybridization	Cloning & sequencing

Required amount of RNA	High	Low

Resolution	Several to 100 bp	Single base

Distinguish Allelic expression?	Limited	Yes

Distinguish splice forms?	Limited	Yes

Discover new genes?	No	Yes

Strandedness?	No	Yes

Dynamic range	Few hundred-fold	> 8000-fold

Reproducibility	Yes	Yes

Cost	Medium	High (due to computation)

The association and integration of historic microarray and RNA-seq data is now an urgent need. This is especially true for cancer centers or institutes with a focus on a particular disease. In these cases, an evolutionary shift in technology may have a disruptive effect, on scientific workflows and associated endeavors, as well as advanced clinical care regimens. Thus, there is a need for disease-based customization built into the advanced bioinformatic methods, which can facilitate in the transition from an older or historic technology (e.g., microarrays) to a new or evolving approach (e.g., RNA-seq) [26,27].

In this study, custom software tools have been developed, some following a model-view-controller (MVC) approach, in order to integrate Affymetric probe set-IDs, and gene annotation information from a variety of sources. Our approach employed a mixture of strategies to maximize the integration, annotation, and association of microarray and RNA-seq datasets. Importantly, our approach is independent of any particular type of transcriptome reconstruction or quantitation tool(s), or file format (e.g. GTF, GFF). Input requirements simply consist of a tab delimited file with column one being an Ensembl Gene ID (if known) and column two being the gene symbol. Additional columns are imported and associated in a record/set-based manner.

For illustrative purposes, output from Cufflinks [28], is directly integrated into a tool pipeline. However, following our tool and file independent design approach, other reconstruction tools such as IsoLasso[29] or TRIP[30], and/or other quantification tools IsoEM[31] or RSEM[32] may be utilized. Finally, multiple myeloma and its associated cancer biology knowledge is utilized and structured to further illustrate the disease-based cross reference operations and annotation strategies, for more effective data culling and exploration. Eventually, we would like to make our software available to the greater scientific community, possibly as open source.

## Methods

### I. Data sets used in this study and database generation

The following files and conversion/association-type annotations were utilized in order to perform proper cross referencing from Affymetrix probe set-IDs to Ensembl gene IDs.

1. Affymetrix annotation for gene cross references (HG-U133_Plus_2 Annotations, CSV format, Release 33 (last update 10/30/12)) was obtained from the Affymetrix website (http://www.affymetrix.com).

2. The Ensembl gene cross references to Affymetrix probe set annotation was obtained using the query interface of Ensembl BioMart (http://www.ensembl.org/biomart/martview/). Key parameters utilized were: Dataset - Homo sapiens genes - GRCh37.p10/ Ensembl Genes 71 and Attributes - Affy HG U133 Plus 2 probe set). Additionally, the Ensembl GTF file (ftp://ftp.ensembl.org/pub/release-71/gtf/homo_sapiens) was also obtained. This file contains Ensembl gene based annotations for corresponding transcripts, as well as chromosomal locations.

3. NCBI gene annotations were obtained from ftp://ftp.ncbi.nlm.nih.gov/gene/DATA/gene2accession.gz, ftp://ftp.ncbi.nlm.nih.gov/gene/DATA/gene_info.gz via the NCBI ftp site, (ftp://ftp.ncbi.nlm.nih.gov/gene/DATA/)

All files were parsed to create a series of look up tables. Subsequently association relationships were further defined and a schema design established. A relational database was created using Microsoft SQL Server 2012 to implement the aforementioned design.

### II. Association algorithms

**Figure 1:**
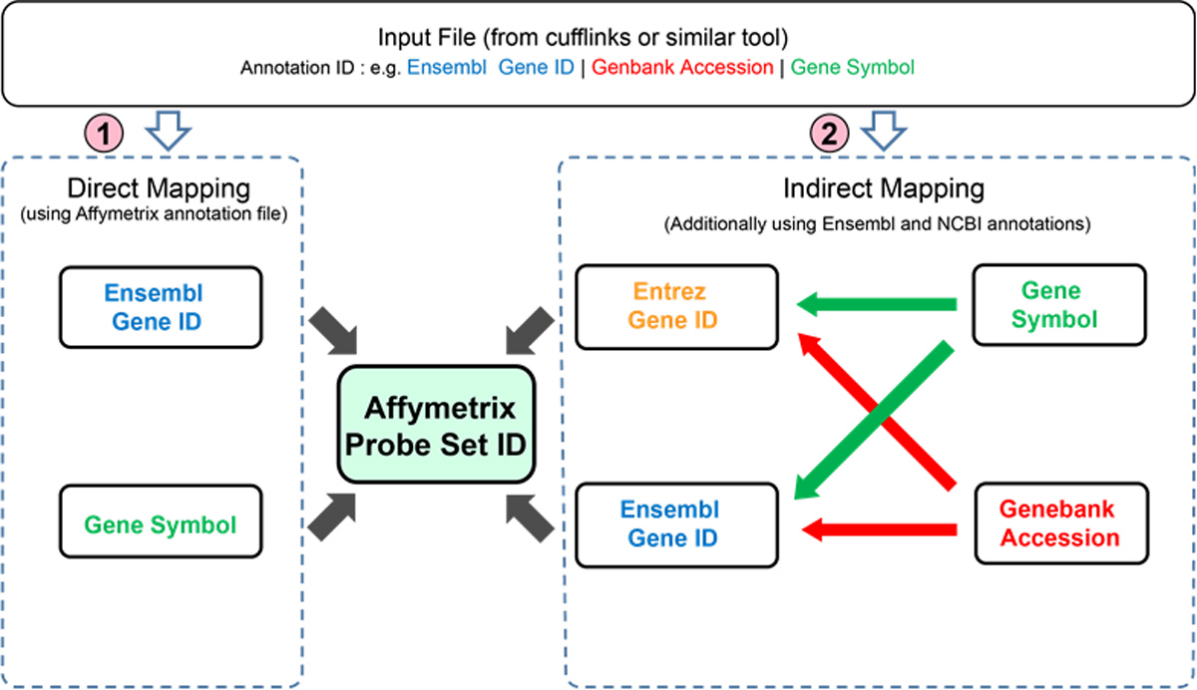
**Affymetrix to NGS association tool algorithm**. Associations between Affymetrix probe setIDs and Ensembl gene identifiers as well as gene symbols are established by either a direct or indirect method.

Associations between Affymetrix probe set-IDs and Ensembl gene identifiers as well as gene symbols were established by either an indirect or direct method. The algorithmic approach is illustrated in Figure 1.

#### Algorithm for direct association

Direct association utilizes cross references available from within the Affymetrix annotation file. This involves first identifying the Affymetrix probe set-ID corresponding to a given Ensembl Gene ID or gene symbol. This is obtained by querying the association look up tables of Ensembl gene to Affymetrix probe set-ID or gene symbol to Affymetrix probe set-ID relationships. For example, given the Ensembl gene ID, "ENSG00009879", a corresponding Affymetrix probe set-ID was searched and identified using a specific SQL query.

#### Algorithm for indirect association

Indirect association involves the use of additional external data sources and a variety of strategies are utilized. We established cross references from NCBI and Ensembl BioMart to enhance our association abilities. This method is automatically invoked when the direct association approach is not successful. The indirect method may first involve using a Genebank Accession and associating to an Entrez Gene ID, then associating the Entrez Gene ID to both an Affy probe set-ID(s) and if possible, also a Gene Symbol (for completeness). Alternatively, a Genebank Accession may be associated to an Ensembl Gene ID, then that Ensembl Gene ID is associated to an Affy probe set-ID(s), and if possible a Gene Symbol. Finally, a gene symbol may be associated either to an Ensembl Gene ID or Entrez Gene ID (depending on success) and then the Ensembl or Entrez Gene ID associated to an Affy probe set-ID(s).

#### Algorithm for locus based association

In locus based association, given a chromosomal location, a set of genes that associate to that specific location can be obtained. This is achieved by querying for an association locus in a table based on the Ensembl annotations. This table contains a chromosomal location and structure for each Ensembl Gene ID.

### III. Interface

**Figure 2:**
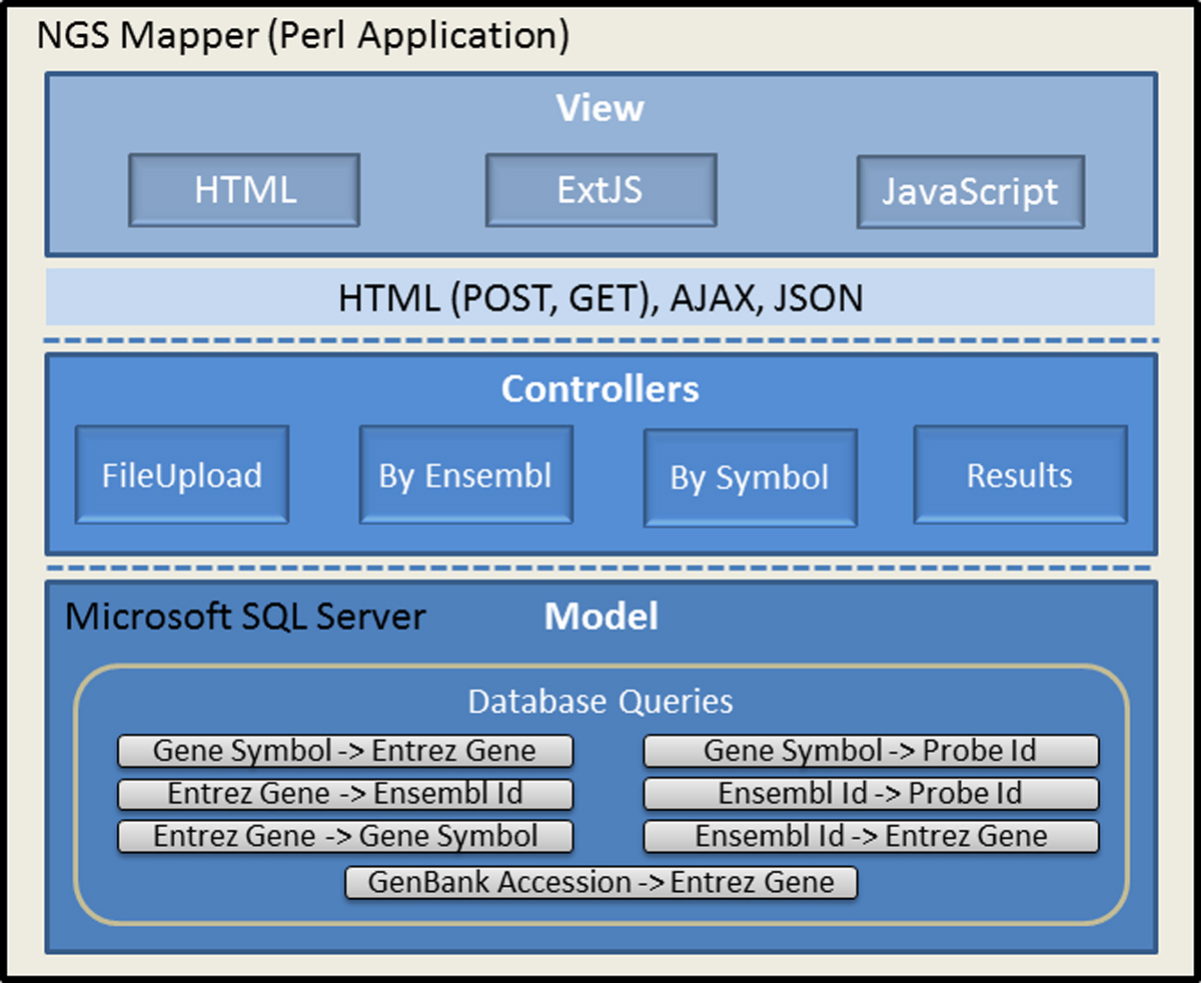
**Design of the NGS association system**. A model-view-controller (MVC) software engineering architecture methodology was utilized to promote code reusability, modularity, and extensibility.

Supported are both browser-based and command line interfaces. The web browser interface design follows a model-view-controller (MVC) approach. Figure 2 illustrates the approach for our system. Here, the display functionality of the interface is separated from the model (database) elements, by using a set of controllers. Using the aforementioned software engineering architectural method promotes code reusability, modularity and extensibility. Thus, future design enhancements, e.g., new features and functions, can be added in an efficient manner.

#### Basic association using web or command line interface

**Figure 3:**
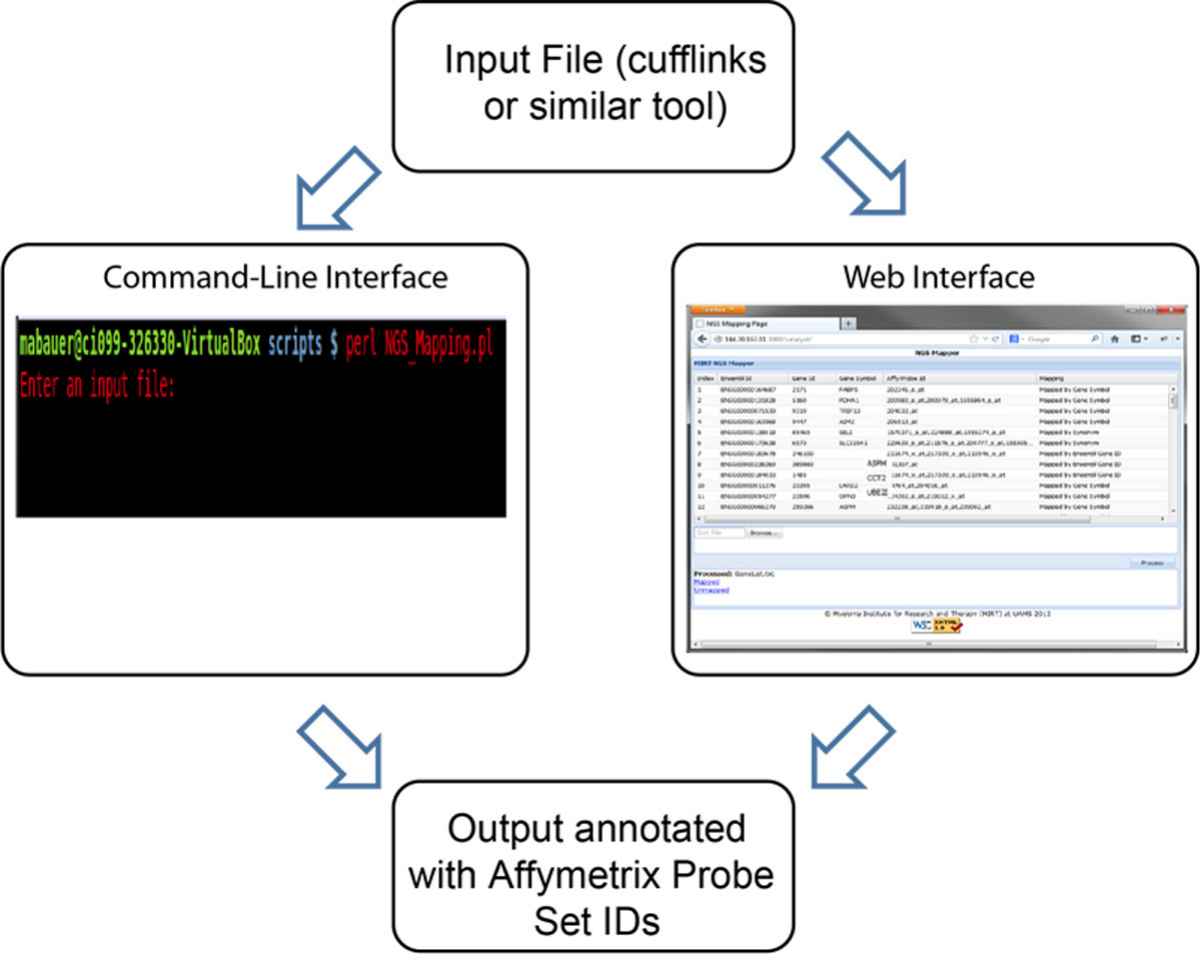
**Interface for the NGS association system**. Both web browser and command line interfaces are supported. The web interface is dynamic and takes as input the output of Cufflinks or similar tool. The NGS association system is independent of any particular transcriptome reconstruction or quantitation tool or file format. Input formatting solely consists of an Ensembl Gene ID in the first column, and gene symbol or gene accession number in the second column, respectively. Additional columns/variables are able to be imported and conveyed in a seamless manner. Using association algorithms, the aim is to link Gene IDs or GeneBank accessions, and Gene symbols with corresponding Affy probe set-ID(s).

The user interface is illustrated in Figure 3. The web interface is dynamic and takes as input the output of Cufflinks, or similar tool. Input formatting consists of an Ensembl Gene ID in the first column, and a gene symbol or gene accession number in the second column, respectively. Additional columns/variables are able to be imported and conveyed in a seamless manner to allow existing data to be further annotated with association data. The aim is to associate the Gene IDs or the GenBank accessions, and Gene symbols with the corresponding Affymetric probe set-ID(s) using the association algorithms. Thus, the gene ID or gene symbol is associated by a direct or indirect method, with the purpose to cross reference an Affymetric probe set-ID. Additional columns of data from Cufflinks or similar tool are conveyed with the data set and incorporated into a relational framework. Finally, connecting the same RNA-seq data with multiple microarray experiments is performed simply by invoking an additional instance of the software, specifying the desired data sets and display/monitor.

A command line interface is also provided. In a standalone manner, the association and cross referencing capabilities are utilized. A single line command specifying the input and output files is required.

#### Cell culture material

The following MM cell lines were used in this study. RPMI 8226 was obtained from ATCC (Cat # CCL-155). H929 was also obtained from ATCC (Cat # CRL-9068). All cell lines were grown in RPMI-1640 medium (ATCC, Cat # 30-2001) with 10% FBS. RNA was extracted using the RNeasy kit (Qiagen) according to manufacturer instructions.

#### Microarray and RNA-seq datasets

**Figure 4:**
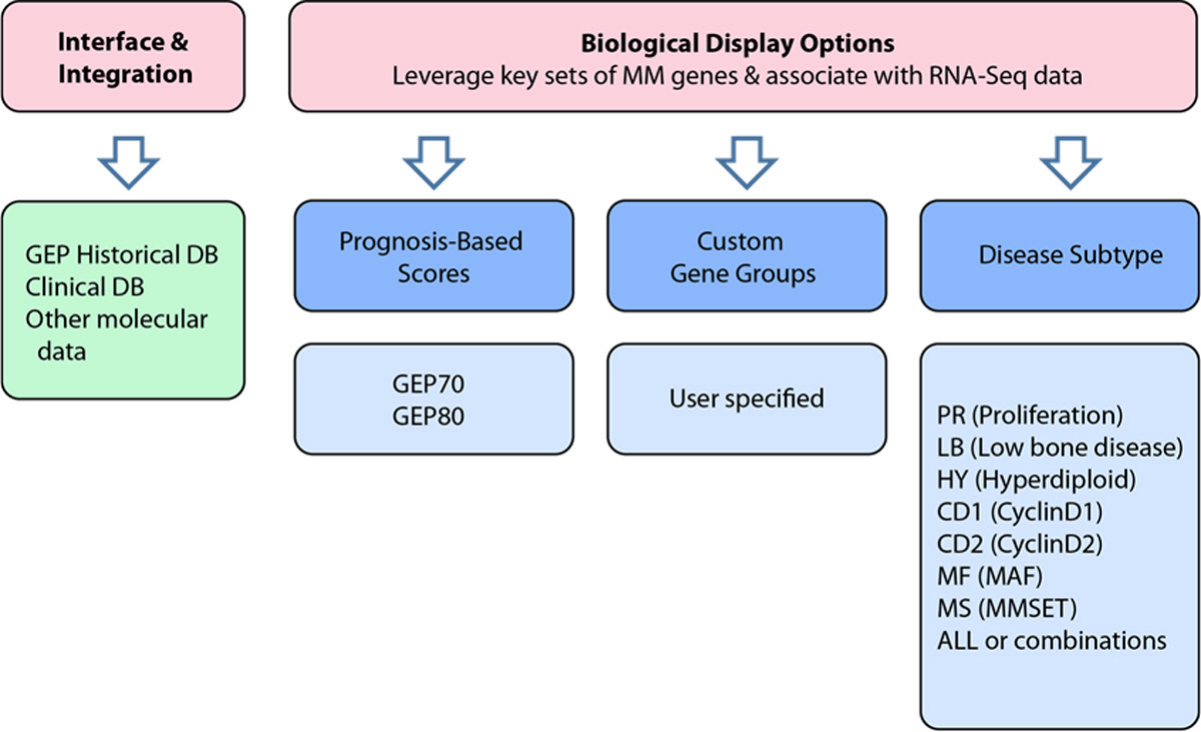
**Interface options for the NGS association system**. A biologically directed filtering through large RNA-seq data sets allows the researcher to quickly leverage and focus on important MM gene and gene isoform subsets, many of which were developed through microarray approaches.

All microarray data files were obtained from the study archive section in our MM historical database. See Figure 4, GEP Historical DB. Microarray datasets were processed as previously reported [33].

For each RNA-seq sample, we prepared Illumina mRNA-seq libraries using the TrueSeq mRNA kit v2. The starting sample material was 100 ng of total RNA and fashioned according to the manufactures instructions. Poly-A selection for mRNA isolation using streptavidin-coated magnetic beads, followed by thermal mRNA fragmentation, was used during the sample prep process. The fragmented mRNA was subjected to cDNA synthesis using reverse transcriptase according to the manufacturer's instructions. The cDNA was then converted into double stranded cDNA followed by an end repair process, and then ligated to Illumina paired end (PE) adaptors. Size selection was performed using AMPure XP beads (Beckman Coulter), generating cDNA libraries ranging in size from 300 to 350 bp (base pairs). Libraries were then enriched using 15 cycles of PCR and purified again by AMPure XP beads. Each library was run at a concentration of 10 pM. All RNA-seq experiments were run on an Illumina HiSeq-2000 in an identical manner utilizing 101 bp PE sequencing. In summary, the fragment length is ~300-350 bp and thus the inner-mate-distance is ~98-148 bp since we are performing 101 bp PE sequencing.

#### RNA-seq read alignment and transcriptome processing

The RNA-seq data analysis utilized the Tuxedo suite. All files/experiments were processed in an identical manner using a standard pipeline protocol. RNA-seq reads for each library were mapped independently using TopHat version 2.0.8 (http://tophat.cbcb.umd.edu/) against the human genome build hg19. HiSeq 2000 libraries were aligned with the following options: "-p 8 -G Ensembl.gtf", where Ensembl.gtf contains the coding transcripts in GTF format. Basic statistics of the two RNA-seq data sets used in this study can be found in additional file 1. Cufflinks version 2.1.1 (http://cufflinks.cbcb.umd.edu/) was run separately for transcriptome reconstruction and quantitation for each generated BAM file with the following options: "-p 8 -g Ensembl.gtf", thus allowing for both annotation and novel transcript discovery.

## Results and discussion

In this study, custom software methodologies have been developed to integrate and cross reference Affymetrix probe set-IDs and gene annotation information from a variety of sources. Given our current and historical work involving microarray technology and disease focus at MIRT (Myeloma Institute for Research and Therapy), a review of key microarray experiments and the cancer biology gleaned was cataloged and studied. Further analyses and computational exercises were then conducted. Methods to best incorporate particular types of cancer biology knowledge gained from microarray studies for leveraging purposes towards RNA-seq, as well as strategies for association, data reduction, display and biological correlation were pursued and continue to be an active research area.

The web interface enables one to associate from Ensembl Gene IDs to Affymetrix probe set-IDs in simple steps. The user can upload as input, a Cufflinks (or similar tool) file, using the 'Browse' button. By clicking the 'Process' button, RNA-seq gene IDs are associated internally. The result is an Affymetrix annotated Cufflinks data file, which is displayed in a panel, as well made available in the form of a downloadable file. Such steps are illustrated in the figure contained in additional file 2. The 'Filter by member list' option is used to reduce a RNA-seq dataset to a smaller list of biologically relevant members, e.g. the 70 probe sets that make up the GEP-70 risk score [11]. This is displayed in additional file 3. Finally, a biologically deeper view of the experimental data is obtained by expanding the gene list to include the corresponding isoform transcripts using the 'isoform tree' option. This is shown in additional file 4. Here an isoform level analysis of the output from the differential expression aspect of Cufflinks (i.e., Cuffdiff) is displayed by our tool for the genes *YWHAZ*, *CCT2 *(note: compressed isoform tree), and *WEE1*.

In addition to association, one can also use or create custom gene lists. Figure 4 illustrates these concepts and features implemented to date. A biologically directed filtering through large RNA-seq datasets is the aim. We have pre-built gene sets for known MM prognostic scoring schemes, namely GEP-70, GEP-80 [33], as well as gene sets for MM molecular subtypes [12]. Thus, researchers can upload their RNA-seq Cufflinks file and then filter their data by any of these criteria. For instance, a GEP-70 selection would result in a filtered list of 70 genes and their corresponding expression values for the RNA-seq genes and isoforms found in the sample input file. Hence, the cancer biologist or clinical researcher can now quickly leverage, target and focus on this important MM subset, which was discovered and developed through microarray approaches.

One of the advantages of RNA-Seq studies versus microarray for transcriptome profiling is its single-base level resolution. This allows expression detection at both a gene and isoform level as opposed to only the gene level for microarrays. Table 2 lists filtered experimental output from a custom gene list composed of eight key MM genes. The cell line H929, which was derived from a 62 year old female with MM, was used in this experiment.

**Table 2:** Filtered experimental data from cell line H929

Gene Name	Gene ID	Transcript ID	RNA-Seq Transcript Expression (FPKM)	RNA-Seq Gene Expression (FPKM)	Representative Affymetrix Probe set Id	Chromosome	Affymetrix Expression
CCND1	ENSG00000110092	ENST00000539241	19.5901	42.7301	208712_at	chr11q13.2	279.6

CCND1	ENSG00000110092	CUFF.2094.1	15.7855	42.7301	208712_at	chr11q13.2	279.6

CCND1	ENSG00000110092	ENST00000227507	7.35445	42.7301	208712_at	chr11q13.2	279.6

**CCND3**	**ENSG00000112576**	**ENST00000372991**	**43.8105**	**43.8105**	**1562028_at**	**chr6p21.1**	**228.4**

DKK1	ENSG00000107984	ENST00000373970	0.765215	1.24334	204602_at	chr10q21.1	521.7

DKK1	ENSG00000107984	CUFF.403.1	0.478122	1.24334	204602_at	chr10q21.1	521.7

**FGFR3**	**ENSG00000068078**	**ENST00000260795**	**25.4733**	**48.3278**	**204380_s_at**	**chr4p16.3**	**870.7**

**FGFR3**	**ENSG00000068078**	**CUFF.20476.3**	**12.4934**	**48.3278**	**204380_s_at**	**chr4p16.3**	**870.7**

**FGFR3**	**ENSG00000068078**	**CUFF.20476.2**	**10.3612**	**48.3278**	**204380_s_at**	**chr4p16.3**	**870.7**

MAF	ENSG00000178573	ENST00000393350	11.1179	23.4381	1566323_at	chr16q23.1	7.1

MAF	ENSG00000178573	CUFF.7714.4	7.70292	23.4381	1566323_at	chr16q23.1	7.1

MAF	ENSG00000178573	ENST00000326043	4.61729	23.4381	1566323_at	chr16q23.1	7.1

**MAFB**	**ENSG00000204103**	**ENST00000373313**	**0.713849**	**0.713849**	**218559_s_at**	**chr20q12**	**141.9**

NFKB1	ENSG00000109320	CUFF.21095.8	7.80674	10.7257	209239_at	chr4q24	977.1

NFKB1	ENSG00000109320	CUFF.21095.12	2.919	10.7257	209239_at	chr4q24	977.1

**WHSC1**	**ENSG00000109685**	**ENST00000508355**	**121.89**	**249.395**	**1557780_at**	**chr4p16.3**	**643.5**

**WHSC1**	**ENSG00000109685**	**CUFF.20684.17**	**68.9562**	**249.395**	**1557780_at**	**chr4p16.3**	**643.5**

**WHSC1**	**ENSG00000109685**	**ENST00000382891**	**58.5494**	**249.395**	**1557780_at**	**chr4p16.3**	**643.5**

Here, the custom MM gene list is composed of: *CCND1, CCND3, DKK1, FGFR3, MAF, MAFB, NFKB1*, and *WHSC1*. The columns "Gene ID" and "Transcript ID" contain the Ensembl designations. Note: if an isoform transcript is novel it will have a "CUFF" prefix. The next two columns contain the respective Fragments Per Kilobase of transcript per Million mapped reads (FPKM) values. The remainder of the table contains a representative Affymetrix probe set-ID, chromosome number and location, and finally the Affy expression value from the associated microarray experiment.

**Figure 5:**
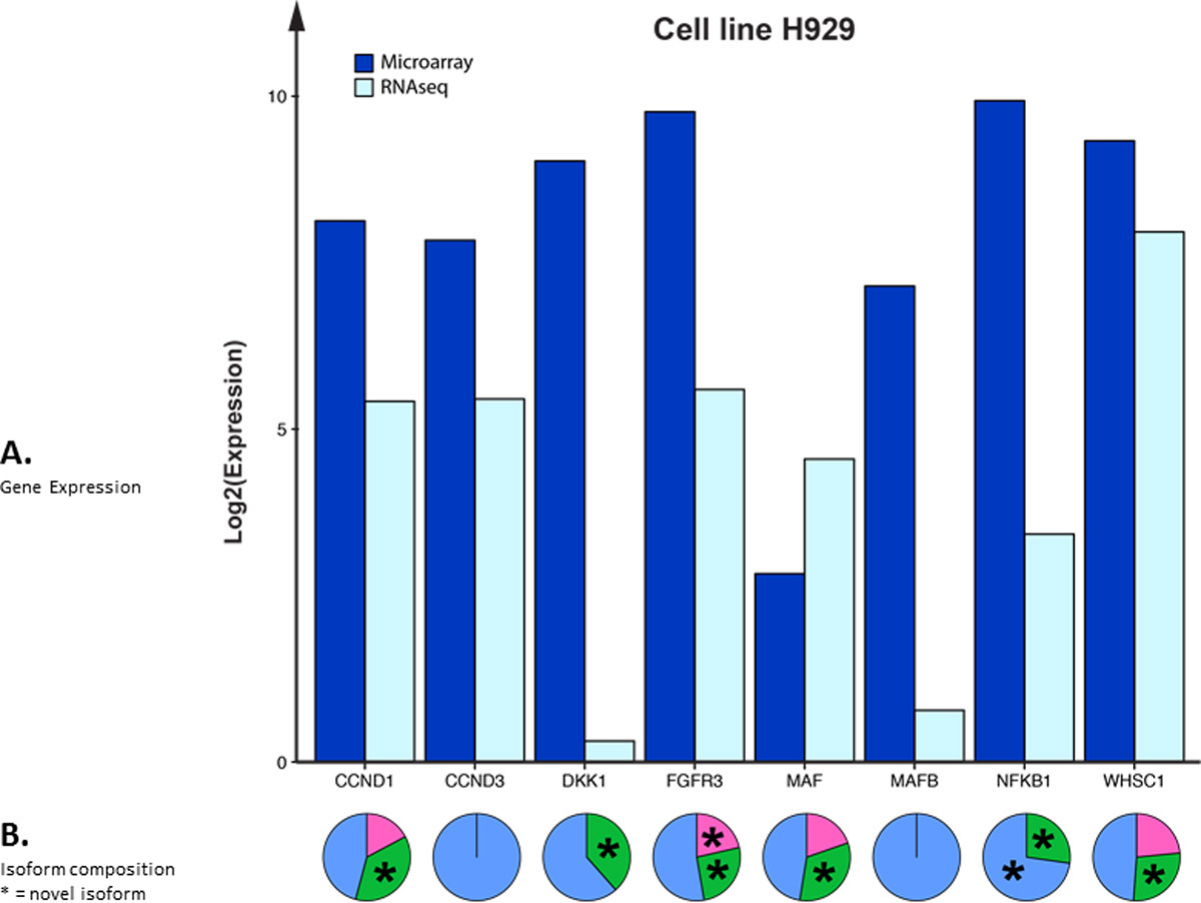
**Visualizing gene and gene isoform expression data in the cell line H929**. This is a system generated graphic illustration of table 2. Section A shows the gene expression values for both the microarray and corresponding RNA-seq experiments. Here, for RNA-seq data, Log2(Expression) corresponds to Log2(FPKM). For microarray data, Log2(Expression) corresponds to the log2 function applied to the normalized absolute intensities from the microarray. Bar plots are used as a visualization aid for the tabular data, and are not meant as a direct comparison between the two different platform technologies. Section B shows an isoform-based comparison, on a gene-by-gene basis, for the RNA-seq gene expression values and allows for an easier identification of dominant forms.

Applying visualization approaches to complex data may aid in speeding analysis, as well as identifying key or subtle trends and patterns. Thus, a continued research effort in our group is to identify data sets amenable to an intuitive graphic representation. Figure 5 is a system generated graphic illustration of table 2. **Section A **shows the gene expression values for both the microarray and corresponding RNA-seq experiments. For RNA-seq data, Log2(Expression) corresponds to Log2(FPKM). For microarray data, Log2(Expression) corresponds to the log2 function applied to the normalized absolute intensities from the microarray. Bar plots are used as a visualization aid for the tabular data, and are not meant as a direct comparison between the two different platform technologies. The log2 approach is based on the publications from Mortazavi et al., and Marioni et al.[25,34]**Section B **shows an isoform-based composition, on a gene-by-gene basis, for the RNA-seq gene expression values. The isoform graphic allows for an easier identification of dominant forms. Novel isoforms are denoted by an asterisk. Even simple programmatic renderings of data, when structured with a proper context, e.g., biological relevance, may aid with analysis in a significant manner.

To further investigate the biological significance of RNA-seq data, an interface to automatically invoke the Integrative Genomics Viewer (IGV)[35] from the browser with experimentally rendered data has been developed. Additional file 5 illustrates this feature. Here, the isoforms for *FGFR3 *are checked/selected for IGV viewing, analysis, etc. By programmatically generating the necessary files required to view specific gene isoform transcripts further analyses may be conducted. For instance, novel and known/annotated isoform transcripts can be graphically compared. Isoforms can also be interrogated to the level of amino acid assignments according to the three possible open reading frames (ORF) provided by IGV. Thus, the relevance of isoforms can now be more easily viewed, compared, and judged for biological significance.

Additional file 6 contains results of an IGV analysis from cell line H929, with gene *DKK1*, and concerns the origin of the novel isoform CUFF.403.1. Note: Dickkopf-related protein 1, which is encoded by the *DKK1 *gene, is associated with osteolytic bone lesions in patients with MM, and is also a known inhibitor of the WNT signaling pathway. Because of this, *DKK1 *and its gene products, are now the subject of active developmental therapeutics drug targeting. Much of this early work was accomplished with microarray technology at MIRT. As shown in table 2 and Figure 5, only two *DKK1 *isoforms were found during the RNA-seq experiment. These are ENST00000373970, which is known and contained in the Ensembl annotation, and CUFF.403.1, which is not known and is thus potentially novel. These two detected isoforms are displayed in the pink region of additional file 6, and the novel isoform (CUFF.403.1) is colored green. In the IGV analysis, all four known *DKK1 *isoform annotations from Ensembl are included along with five red arrows, which serve to illustrate the origin of the various coding segments comprising the novel isoform CUFF.403.1. Thus in this case, the origin of this discovered isoform appears to be the result of a new alternative splicing of the mRNA for gene *DKK1*. This potentially exciting finding was greatly aided by prior MM microarray studies.

**Figure 6:**
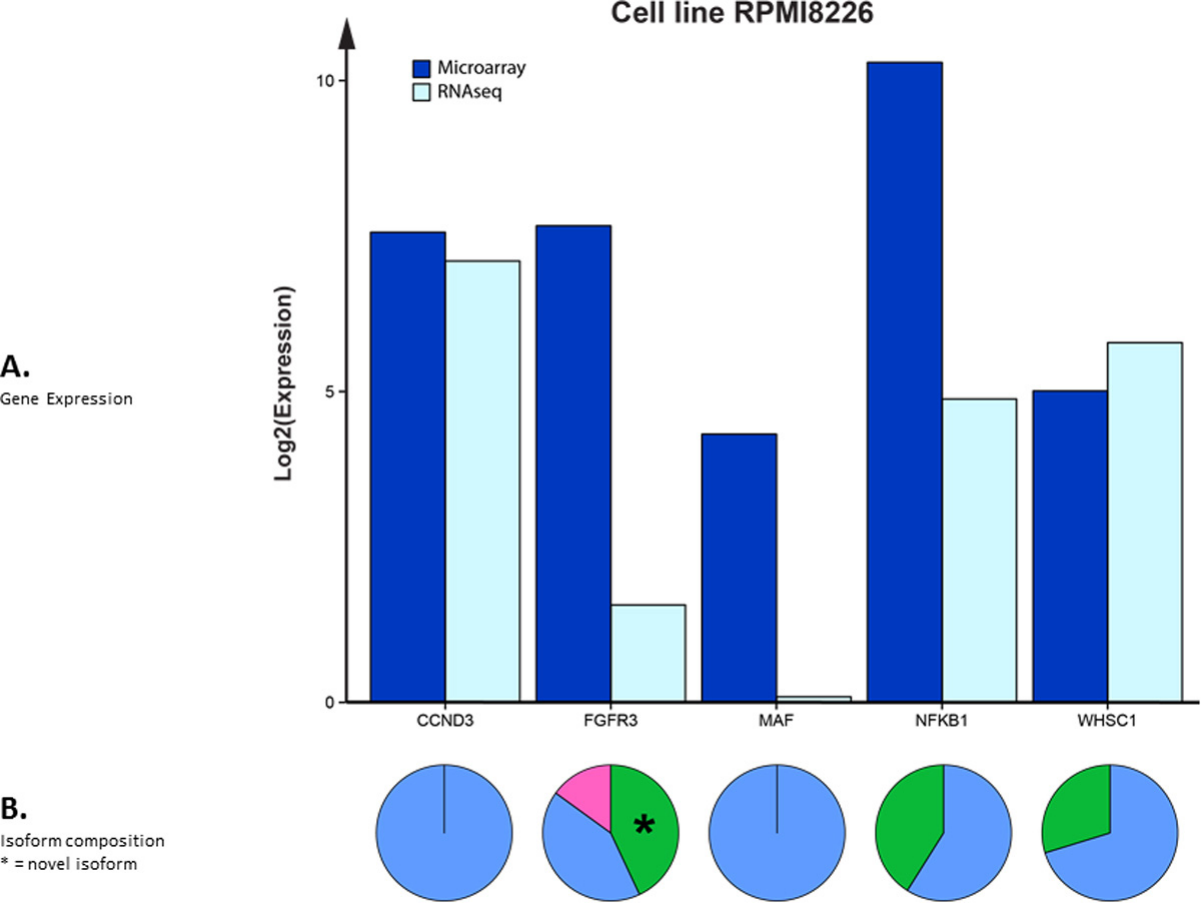
**Visualizing gene and gene isoform expression data in the cell line RPMI-8226**. This is a system generated graphic illustration of table 3. All computations and comparisons were performed in an identical manner as in figure 5.

A second combined RNA-seq with microarray experiment was performed utilizing the cell line RPMI 8226. This cell line was derived from a 61 year old male diagnosed with MM. The same eight key MM genes were used and table 3 lists the filtered results. Only transcripts from five of the eight genes were found in this particular RNA-seq experiment. Figure 6 displays a computed graphic representation of the gene expression values (**section A**) for microarray and RNA-seq. A graphic illustration of isoform contributions is contained in **section B**. All computations and comparisons were performed in an identical manner as in Figure 5. Although the Affymetrix signal from *FGFR3 *is a bit low, it was chosen for illustrative purposes.

**Table 3:** Filtered experimental data from cell line RPMI-8226

Gene Name	Gene ID	Transcript ID	RNA-Seq Transcript Expression (FPKM)	RNA-Seq Gene Expression (FPKM)	Representative Affymetrix Probe set Id	Chromosome	Affymetrix Expression
CCND3	ENSG00000112576	ENST00000372991	137.068	137.068	1562028_at	chr6p21.1	188.9

**FGFR3**	**ENSG00000068078**	**CUFF.23217.9**	**1.27549**	**2.96453**	**204380_s_at**	**chr4p16.3**	**202.9**

**FGFR3**	**ENSG00000068078**	**ENST00000340107**	**1.24395**	**2.96453**	**204380_s_at**	**chr4p16.3**	**202.9**

**FGFR3**	**ENSG00000068078**	**ENST00000260795**	**0.445095**	**2.96453**	**204380_s_at**	**chr4p16.3**	**202.9**

MAF	ENSG00000178573	ENST00000393350	0.00202724	0.00202724	1566323_at	chr16q23.1	19.9

**NFKB1**	**ENSG00000109320**	**ENST00000505458**	**17.3606**	**29.4494**	**209239_at**	**chr4q24**	**1253.8**

**NFKB1**	**ENSG00000109320**	**ENST00000226574**	**12.0888**	**29.4494**	**209239_at**	**chr4q24**	**1253.8**

WHSC1	ENSG00000109685	ENST00000382891	38.771	55.1773	1557780_at	chr4p16.3	32.2

Additional file 7 contains results of an automatically invoked IGV analysis from cell line RPMI-8226, gene *FGFR3*, and the three identified isoforms identified during the RNA-seq experiment. **Section A **shows a graphic comparison the two known/annotated Ensembl isoforms (ENST00000340107 and ENST00000260795) along with the discovered/novel isoform CUFF.23217.9. The unique coding segment of CUFF.23217.9 is noted by the red brace, and graphically its uniqueness is apparent. Thus, step two of the analysis concerns biological relevance. An interrogation of the amino acids that may be generated from the unique coding segment of CUFF.23217.9 is shown in **section B**. In each of the three ORFs, stop codons are encountered.

If a novel isoform generates a stop codon early in the corresponding amino acid chain, the protein viability is highly unlikely. This is due to the nonsense-mediated mRNA decay pathway. This may be contrary to intuition, that the predominant consequence of nonsense mutations is not the synthesis of truncated proteins. Rather, these nonsense transcripts are recognized by the cell and efficiently degraded [36]. Since stop codons are occurring early in the protein sequence in all ORFs for CUFF.23217.9, it is not biologically significant. Thus, RNA-seq is serving to verify the impact of these nonsense mutations.

## Conclusions

There is much to learn about the complexity of cancer genomes. As technology advances, it will be critical to continue to leverage what has been gained from historic platform technologies (e.g., microarrays) to new approaches (RNA-seq). Thus in addition to the requisite bioinformatics-based migration due to technical aspects, custom approaches, which harness particular salient aspects of the biology of a disease, may be of assistance. In this study, we have begun to lay a framework towards such endeavors for MM. Specifically, through the creation and development of novel bioinformatic approaches to aid in the visualization and facilitation of both annotation and association of historic microarray data in conjunction with richer RNA-seq data. It is our hope that such efforts will assist with our understanding of which genes are mutated, the pathways impacted by these mutations, and how these data inform our models of MM cancer biology, so as to ultimately improve the outcomes of our patients.

## Competing interests

The authors declare that they have no competing interests.

## Authors' contributions

DJJ and SSC conceived and designed the study. SSC, EAP, MAB, CJH and DJJ performed experiments and analyses. MAB, SSC, and EAP implemented the software. All authors wrote and approved the manuscript.

## Supplementary Material

Additional file 1 **Basic statistics for RNA-seq data sets**. Click here for file

Additional file 2 **Initial loading and mapping of a Cufflinks data file**. The association of Ensembl Gene IDs to Affymetrix probe set-IDs can be accomplished via simple steps by the web interface. A Cufflinks (or similar tool) file may be uploaded as input via the "Browse" button. By pressing the "Process" button, RNA-seq gene IDs are associated internally. The Affymetrix annotated Cufflinks data file can then be display in a panel and additionally be downloaded as a file. Click here for file

Additional file 3 **Filtering the Cufflinks data file by the GEP-70 gene list**. The "Filter by member list" option is used to reduce a RNA-seq dataset to a smaller list of biologically relevant members, in this case, the 70 probe sets that make up the GEP-70 risk score. Click here for file

Additional file 4 **Viewing Cufflinks (Cuffdiff) data at the isoform level**. A biologically deeper view of the experimental data is possible by expanding the gene list to include corresponding isoform transcripts via the "isoform tree" option. Click here for file

Additional file 5 **Interface for automatic invocation of IGV**. An interface to automatically invoke the Integrative Genomics Viewer (IGV) from the NGS browser with experimentally rendered data was developed. This facilitates further investigations regarding the biological relevance of RNA-seq data streams. Click here for file

Additional file 6 **The origin of novel isoform CUFF.403.1 from gene *DKK1 *and cell line H929**. Two *DKK1 *isoforms were found during the RNA-seq experiment and are contained in the pink region of the graphic image. They are ENST00000373970, which is known and contained in the Ensembl annotation, and CUFF.403.1, which is not known and is thus potentially novel. The novel isoform is colored green. In IGV analysis, all four known *DKK1 *isoform annotations from Ensembl are included along with five red arrows, which serve to illustrate the origin of the various coding regions comprising novel isoform CUFF.403.1. Here, the origin of this discovered isoform appears to be the result of a new alternative splicing of the mRNA for gene *DKK1*. Click here for file

Additional file 7 **Three identified isoforms for gene *FGFR3 *from cell line RPMI-8226**. An automatically invoked IGV analysis from cell line RPMI-8226, gene *FGFR3 *reveals the three identified gene isoforms. Section A displays the graphic comparison of the two known/annotated Ensembl isoforms (ENST00000340107 and ENST00000260795) along with the discovered/novel isoform CUFF.23217.9. The unique coding segment of CUFF.23217.9 is noted by a red brace and labeled as a novel exon. Section B contains the second step of the analysis, specifically, the biological relevance of the novel exon. Here an interrogation of the amino acids from each of the three open reading frames reveals stop codons. As a result, the biological relevance of the novel exon is not significant due to the likely activation of the nonsense-mediated mRNA decay pathway, and thus no protein viability. Click here for file

## Declarations

Publication of this work was supported by Program Project Grant CA 55813, from the National Institutes of Health (NIH), National Cancer Institute (NCI), Bethesda, MD, USA.

This article has been published as part of *BMC Bioinformatics *Volume 14 Supplement 14, 2013: Proceedings of the Tenth Annual MCBIOS Conference. Discovery in a sea of data. The full contents of the supplement are available online at http://www.biomedcentral.com/bmcbioinformatics/supplements/14/S14.
