# Supplementary figures and images for: Towards the integration, annotation and association of historical microarray experiments with RNA-seq
Source: BMC Bioinformatics. 2013 Oct 9;14(Suppl 14):S4. doi: 10.1186/1471-2105-14-S14-S4 (PMC3851429; doi:10.1186/1471-2105-14-S14-S4)

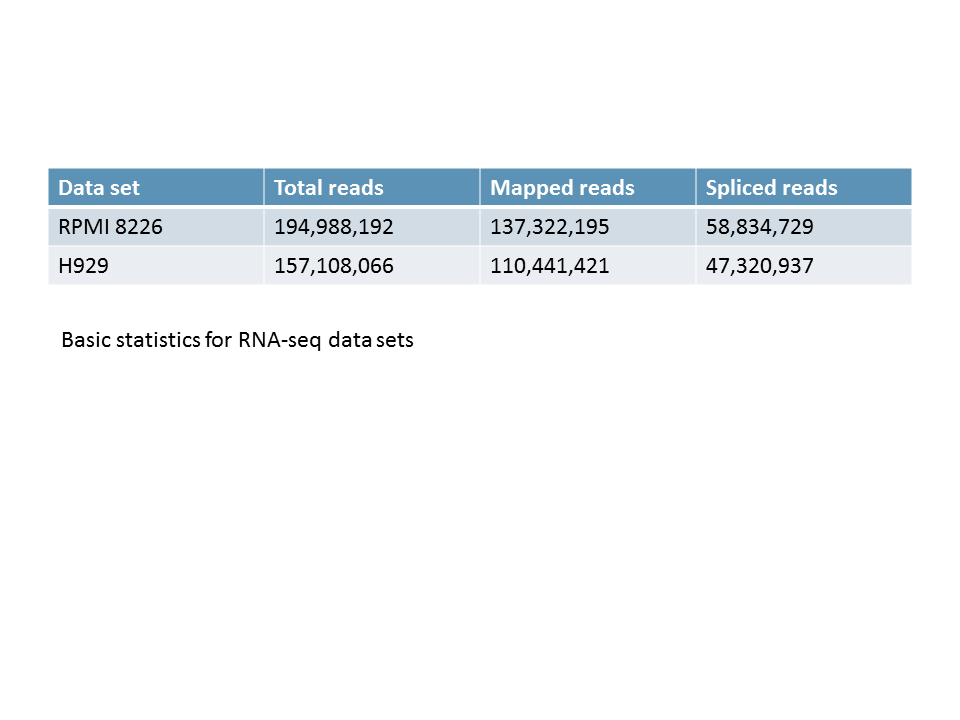

Supplement: Additional file 1 — Basic statistics for RNA-seq data sets. [file 1471-2105-14-S14-S4-S1.tif]

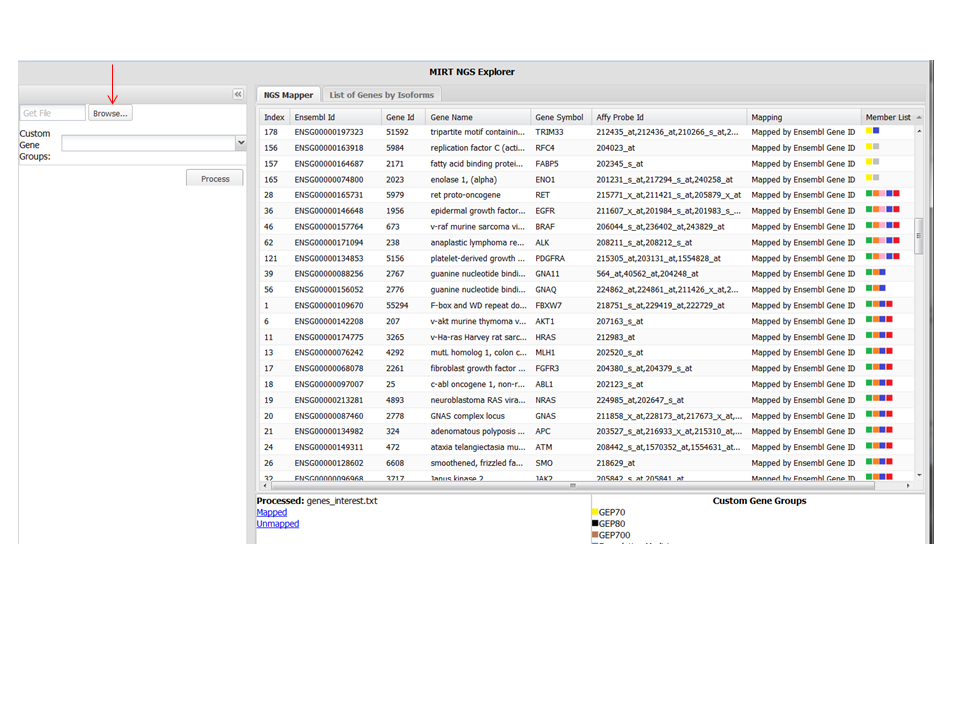

Supplement: Additional file 2 — Initial loading and mapping of a Cufflinks data file. The association of Ensembl Gene IDs to Affymetrix probe set-IDs can be accomplished via simple steps by the web interface. A Cufflinks (or similar tool) file may be uploaded as input via the "Browse" button. By pressing the "Process" button, RNA-seq gene IDs are associated internally. The Affymetrix annotated Cufflinks data file can then be display in a panel and additionally be downloaded as a file. [file 1471-2105-14-S14-S4-S2.tif]

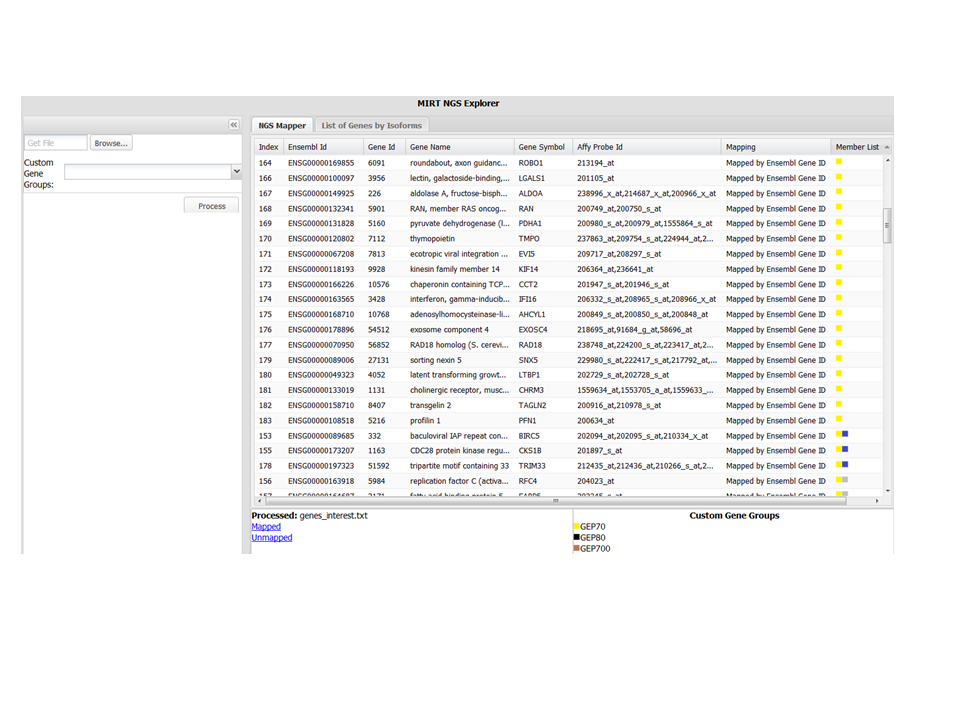

Supplement: Additional file 3 — Filtering the Cufflinks data file by the GEP-70 gene list. The "Filter by member list" option is used to reduce a RNA-seq dataset to a smaller list of biologically relevant members, in this case, the 70 probe sets that make up the GEP-70 risk score. [file 1471-2105-14-S14-S4-S3.tif]

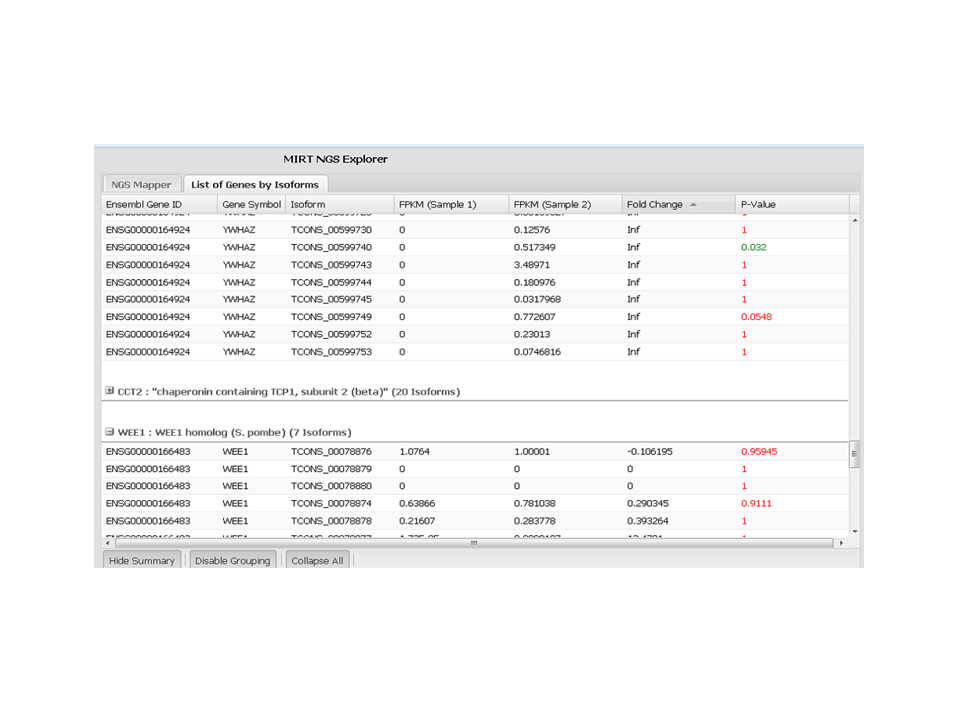

Supplement: Additional file 4 — Viewing Cufflinks (Cuffdiff) data at the isoform level. A biologically deeper view of the experimental data is possible by expanding the gene list to include corresponding isoform transcripts via the "isoform tree" option. [file 1471-2105-14-S14-S4-S4.tif]

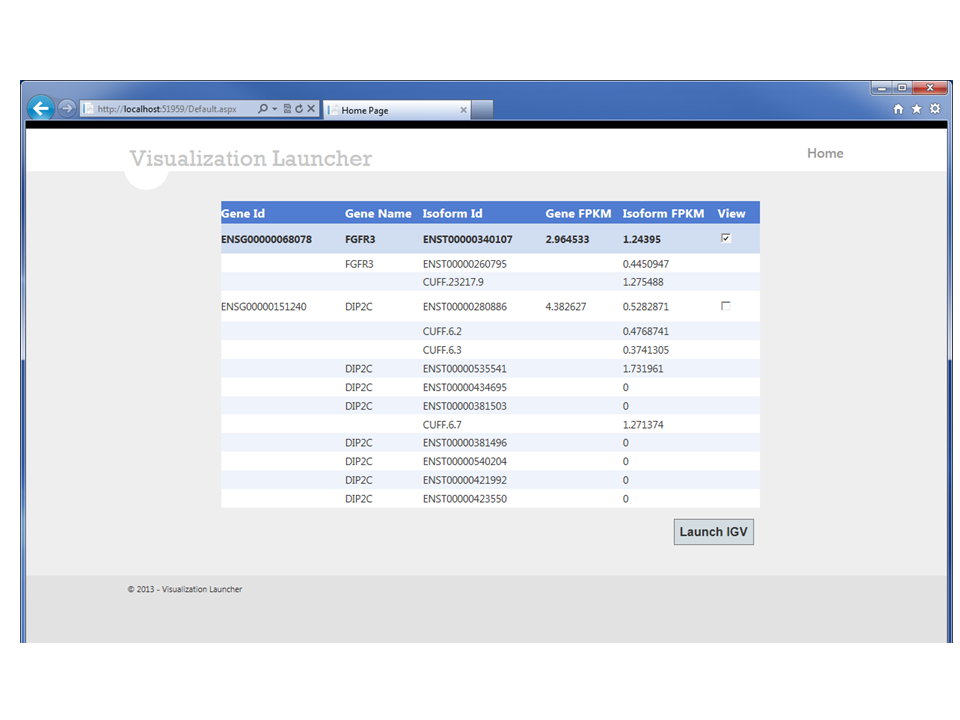

Supplement: Additional file 5 — Interface for automatic invocation of IGV. An interface to automatically invoke the Integrative Genomics Viewer (IGV) from the NGS browser with experimentally rendered data was developed. This facilitates further investigations regarding the biological relevance of RNA-seq data streams. [file 1471-2105-14-S14-S4-S5.tif]

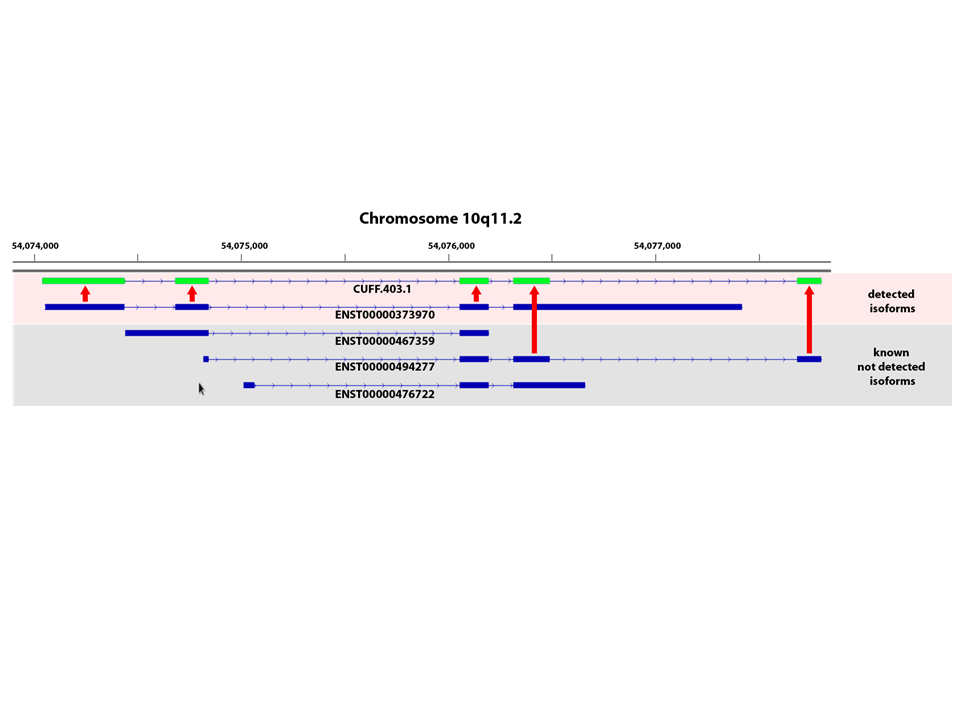

Supplement: Additional file 6 — The origin of novel isoform CUFF.403.1 from gene DKK1 and cell line H929. Two DKK1 isoforms were found during the RNA-seq experiment and are contained in the pink region of the graphic image. They are ENST00000373970, which is known and contained in the Ensembl annotation, and CUFF.403.1, which is not known and is thus potentially novel. The novel isoform is colored green. In IGV analysis, all four known DKK1 isoform annotations from Ensembl are included along with five red arrows, which serve to illustrate the origin of the various coding regions comprising novel isoform CUFF.403.1. Here, the origin of this discovered isoform appears to be the result of a new alternative splicing of the mRNA for gene DKK1. [file 1471-2105-14-S14-S4-S6.tif]

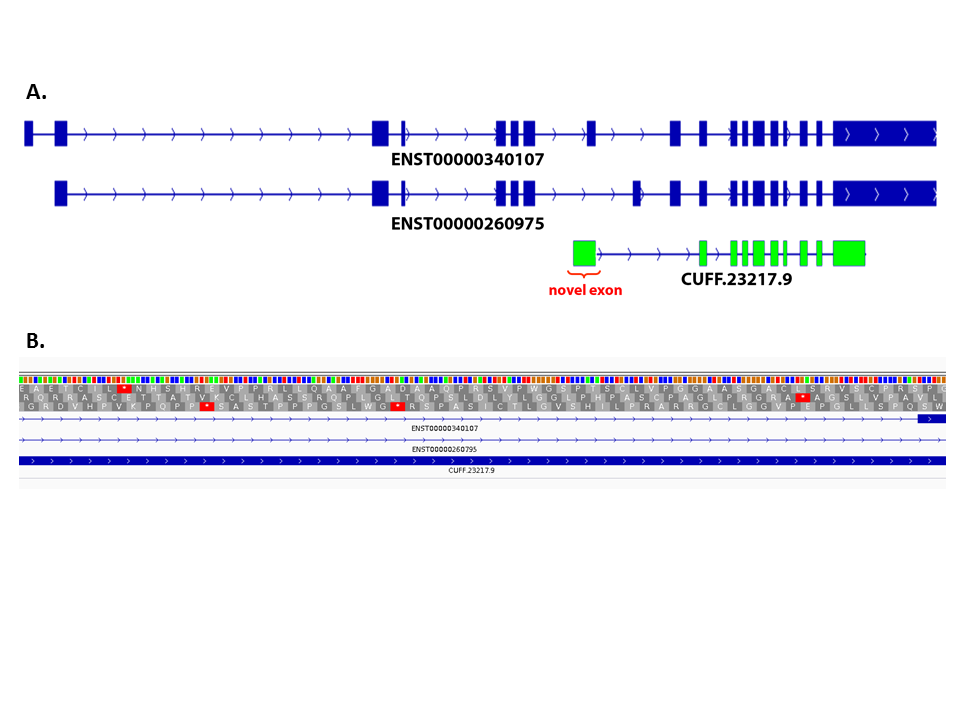

Supplement: Additional file 7 — Three identified isoforms for gene FGFR3 from cell line RPMI-8226. An automatically invoked IGV analysis from cell line RPMI-8226, gene FGFR3 reveals the three identified gene isoforms. Section A displays the graphic comparison of the two known/annotated Ensembl isoforms (ENST00000340107 and ENST00000260795) along with the discovered/novel isoform CUFF.23217.9. The unique coding segment of CUFF.23217.9 is noted by a red brace and labeled as a novel exon. Section B contains the second step of the analysis, specifically, the biological relevance of the novel exon. Here an interrogation of the amino acids from each of the three open reading frames reveals stop codons. As a result, the biological relevance of the novel exon is not significant due to the likely activation of the nonsense-mediated mRNA decay pathway, and thus no protein viability. [file 1471-2105-14-S14-S4-S7.tif]
